# A Monitoring System for Online Fault Detection and Classification in Photovoltaic Plants

**DOI:** 10.3390/s20174688

**Published:** 2020-08-20

**Authors:** André Eugênio Lazzaretti, Clayton Hilgemberg da Costa, Marcelo Paludetto Rodrigues, Guilherme Dan Yamada, Gilberto Lexinoski, Guilherme Luiz Moritz, Elder Oroski, Rafael Eleodoro de Goes, Robson Ribeiro Linhares, Paulo Cézar Stadzisz, Júlio Shigeaki Omori, Rodrigo Braun dos Santos

**Affiliations:** 1 LIT—Laboratory of Innovation and Technology in Embedded Systems and Energy, Universidade Tecnológica Federal do Paraná-UTFPR, 80230-901 Curitiba, PR, Brazil; claytoncosta@alunos.utfpr.edu.br (C.H.d.C.); marcelorodrigues@alunos.utfpr.edu.br (M.P.R.); yamada@alunos.utfpr.edu.br (G.D.Y.); gilberto.2015@alunos.utfpr.edu.br (G.L.); moritz@utfpr.edu.br (G.L.M.); oroski@utfpr.edu.br (E.O.); rgoes@utfpr.edu.br (R.E.d.G.); linhares@utfpr.edu.br (R.R.L.); stadzisz@utfpr.edu.br (P.C.S.); 2 COPEL-Companhia Paranaense de Energia, 82305-100 Curitiba, PR, Brazil; julio.omori@copel.com (J.S.O.); rodrigo.braun@copel.com (R.B.d.S.)

**Keywords:** embedded systems, fault classification, fault detection, monitoring systems, PV plants

## Abstract

Photovoltaic (PV) energy use has been increasing recently, mainly due to new policies all over the world to reduce the application of fossil fuels. PV system efficiency is highly dependent on environmental variables, besides being affected by several kinds of faults, which can lead to a severe energy loss throughout the operation of the system. In this sense, we present a Monitoring System (MS) to measure the electrical and environmental variables to produce instantaneous and historical data, allowing to estimate parameters that ar related to the plant efficiency. Additionally, using the same MS, we propose a recursive linear model to detect faults in the system, while using irradiance and temperature on the PV panel as input signals and power as output. The accuracy of the fault detection for a 5 kW power plant used in the test is 93.09%, considering 16 days and around 143 hours of faults in different conditions. Once a fault is detected by this model, a machine-learning-based method classifies each fault in the following cases: short-circuit, open-circuit, partial shadowing, and degradation. Using the same days and faults applied in the detection module, the accuracy of the classification stage is 95.44% for an Artificial Neural Network (ANN) model. By combining detection and classification, the overall accuracy is 92.64%. Such a result represents an original contribution of this work, since other related works do not present the integration of a fault detection and classification approach with an embedded PV plant monitoring system, allowing for the online identification and classification of different PV faults, besides real-time and historical monitoring of electrical and environmental parameters of the plant.

## 1. Introduction

The growth of renewable energy (RE) sources has been increasingly significant in the last decade. For instance, in 2018, without considering large hydro-power plants, an outstanding amount of 190 GW total new capacity installed was reached worldwide, being 55% of the total power capacity that was installed during that year [1]. Among most frequent renewable sources, solar photovoltaic (PV) has shown the largest growth and it is responsible for around 39% of installed capacity over the course of 2018 [1]. Nevertheless, there are several performance issues that must be considered to increase PV performance. In the context of this work, for instance, PV performance can be compromised due to the high exposure to different weather conditions, like soiling [2] and temperature [3], resulting in electrical and mechanical faults, like cracked cells and short-circuits [4].

With such high exposure, the need of methods to maintain performance, reduce revenue losses and downtime, and ensure rapid fault detection, classification, location, and mitigation in PV systems emerge [5]. One way to achieve that is to include a MS in the PV plant that measures electrical and meteorological variables, manages plant operations (e.g., remote access), detects malfunctions and errors, and reports performance and benchmarking, locally or remotely, through a communication system to the grid operator [6,7]. However, only the MS is not enough to completely solve the problem [8], since PV faults demand specific techniques to detect and classify them, using monitored data [9,10].

Techniques are normally divided into the detection and classification of PV faults, mainly focused on the most recurrent ones, such as open-circuit, short-circuit, and module mismatch [11], in order to accomplish those tasks. In terms of fault detection, there has been several proposals in the literature. In [12], for instance, fault detection that is based on satellite data is proposed. In other work [13], the PV module fault detection using thermal images allied with Canny edge detector is presented. Recently, several methods based on the modeling of PV systems have been proposed [14,15,16], achieving state-of-the-art results in real PV plants. However, such recent models are mostly based on static models, discarding relevant dynamic modeling, and making it difficult to detect events that occur in short intervals of time [17].

In the fault classification context, there are different approaches, such as visual methods [18], thermographic image analysis methods [13], and mathematical methods using theoretical and simulated models of PV plants [19]. More recently, some machine learning-based techniques have been proposed, improving the classification performance in different cases, mainly including the shadowing and degradation of PV modules [9,20,21]. Notwithstanding, most of the methods are focused on simulated data and the authors do not present an extensive analysis of methods for online fault classification.

The limitations that are presented for detection and classification methods can be added to the fact that most of the models do not include results of the solution in a dedicated hardware or system, integrated to a monitoring system, and when they do, the PV plant power output is limited or the detection can only be performed disturbing the normal operation of the system. In this sense, the main contribution of this work is the integration of a fault detection and classification approach with an embedded PV plant monitoring system, allowing for non-intrusive online identification and classification of different PV faults, besides providing a MS integrated to the plant. Additionally, we present a detailed comparison of dynamic models and machine learning approaches to detect and classify, respectively, several real fault scenarios in a 5 kW PV plant, pointing to the most suitable model for online fault detection and classification in PV systems. To the extent of our knowledge, such a proposal has not yet been presented in the related literature.

This paper is organized, as follows. Section 2 addresses related works in terms of monitoring systems, fault detection and fault classification, together with a discussion regarding faults in PV systems. Section 3 describes, in detail, the proposed monitoring system. Section 4 shows the simulated and real datasets used to validate the detection and classification methods proposed in this work. Section 5 and Section 6 present theoretical and methodological aspects of the proposed detection and classification methods, respectively. Section 7 presents the results of the monitoring system, detection, and classification approaches, comparing them with other related works. Finally, Section 8 reports the general conclusions and suggests future research directions.

## 2. Related Works

In order to present the main contributions of this work, we decided to divide the presentation of related works into four main Subsections, which are the basis of this work. Initially, faults in PV systems are discussed, emphasizing the most relevant faults and describing the characteristics of each fault. In the sequence, monitoring systems, fault detection, and fault classification are presented, showing the main limitations observed in the recent literature. Finally, in Section 2.5, the original aspects of this work are detailed with respect to related monitoring systems, fault detection, and classification methods.

### 2.1. Faults in PV Systems

An important point for the evaluation of fault occurrence, as well as its impact, is the survey and details of, at least, the most common faults in photovoltaic systems. In this context, an extensive study on those faults is presented in [22], dividing them into two categories: faults on the direct current (DC) side and faults on the alternating current (AC) side. Faults on the AC side are due to problems with the inverter of the system or the power grid itself. Faults on the DC side are more numerous and they include: problems with the Maximum Power Point Tracking (MPPT) algorithm, faults in the bypass diode, ground fault, arc faults, cell or module mismatch (which may be temporary or permanent), open circuit, and short-circuit. In the context of this work, we selected the most recurrent faults, which includes module mismatch, open-circuit, and short-circuit [11].

Open-circuit fault occurs when, at some point in the system, there is a disconnection, causing the circulation of electrical current to be interrupted. From the power generation point of view, this fault is the one with the greatest impact [23], since it can affect from a single string of modules, up to the entire system, depending on the location of the disconnection and topology of the PV system.

The mismatch of cells or modules occurs when there are cells or modules in the PV system with electrical properties that are very different from the others, impairing their functioning [24]. Mismatch fault can be divided into two subcategories: temporary and permanent. Temporary mismatch is normally caused by events, such as the deposition of dust or snow and by shadowing that is caused by buildings or transmission lines. The permanent mismatch occurs due to the degradation and damage of the affected cells and modules. In the present work, both cases are taken into account, using shadowing and degradation as cases of temporary and permanent mismatch, respectively, both occurring at the module level.

The short-circuit fault occurs when a low impedance path appears along the system. In the case of PV systems, this can occur at several points, such as between two terminals of the same module, two points of the same string, two different strings, and a string with the ground. In this work, the occurrence between two points of the same string will be taken into account, more particularly between the negative terminal of a module and the positive terminal of its adjacent module, which is generally the most impacting short-circuit fault from the energy production point of view.

### 2.2. Monitoring Systems

In grid-connected PV systems, which is the main focus of this work, the variables commonly measured to detect and classify faults are [19,23]: total irradiance; wind speed; wind direction; output voltage and current of each PV array; output power and energy of each PV array; ambient temperature; PV module temperature; grid voltage; bidirectional current of the grid; bidirectional power; and, energy of the grid. Besides allowing the identification of faults in the components of the plant (modules, connection lines, converters, and inverters), these variables are the basis to enable the evaluation of the plant performance in real-time and the improvement of the system reliability, as suggested in [25].

In this sense, several works have been proposed to present data acquisition systems in order to measure those variables. In [7], for instance, the data acquisition is composed of wireless sensors that are distributed around the plant, which measure electrical and meteorological parameters. The authors analyze the performance of the system in a 400 kW transformation center, presenting the results of the sensor network under different conditions of operation. However, fault detection and classification are not discussed in that work, since the main contribution presented by authors is a high-precision protocol for synchronizing all data.

Still, in the context of wireless sensor networks (WSNs), a Zigbee (Zigbee is wireless communication system based on the IEEE 802.15.4 specification for personal area networks using low-power digital radios [26])-based online MS of a grid-connected utility-scale PV system was proposed in [27]. The system measures PV temperature, irradiation, and power output. Additionally, the authors present a web-application, similar to the solution proposed in [28], allowing fault detection and location in real-time. Nevertheless, a common limitation of those works is the high dependence on the correct functioning of the distributed sensors. The failure of one or more sensors compromises the identification and location of faults throughout the plant.

Different approaches have been proposed with the aim of providing a low-cost sensor network for a massive monitoring in a PV plant, and avoiding the high dependence on individual sensors. In [6], for instance, a set of low-cost voltage, current, and temperature sensors was applied in the context of detecting critical faults, such as temporary and permanent shadowing, dirtying, and anomalous aging in a single PV panel. However, short-circuits and other types of faults are not discussed in that work. Besides, the proposed sensor network was installed in a single PV panel and it was not expanded to a PV plant.

Similarly, the development of low-cost sensors was proposed in [29,30], with the aim of obtaining the lowest possible cost for monitoring electrical and temperature variables. Following a different method for data transmission, but keeping the low-cost strategy, in [31], the PV panel’s voltage, current, and temperature were measured and transferred to the central control system using power lines carriers communications (PLC) technology on DC power lines. Similarly, in [32], a system composed of a WSN to obtain information of solar panels for timely repair and maintenance is presented, particularly designed for domestic applications. Nevertheless, because these works are mainly focused on low-cost monitoring systems, they do not present an analysis of faults and malfunctioning of the PV plant—i.e., the use of monitored variables in the context of plant maintenance.

### 2.3. Fault Detection

In general, fault detection for PV systems is based on the modeling of the system in order to compare the results from modeling with real-acquired data, indicating a fault event every time the difference between modeling and acquired data is above some predefined threshold [16]. The modeling step is normally divided into dynamic or static. Static models do not consider time as an independent variable and, due to that, they are normally referred as non-memory models. Dynamic models, on the other hand, take time variations in the model into account. In PV modeling, static models are the most recurrent, in which PV cells are represented by the Single Diode Model [33].

In [5,34,35], a static model that is based on a single diode model is considered in the modeling process to detect faults and predict energy production. However, the main limitation of this group of models is the representation of a generic and static PV cell. By simplifying the PV cell to a generic and static one, individual characteristics and the dynamics of different PV systems may be disregarded, compromising the modeling of certain phenomena and, consequently, the identification of faults that occur in short intervals of time. Nevertheless, from the diagnosis point of view, the static model is appropriate for detecting aging and degradation issues, due to the long-term characteristics of such faults [35].

In [14,15], a statistical approach based on the multivariate exponentially weighted moving average charts is proposed for fault detection in order to improve those limitations of single-diode models. The authors generated array’s residuals of DC current, voltage and power, considering temperature and irradiance as inputs. With the residuals, it is possible to calculate the difference between the measurements and the predictions for the electrical variables from the single-diode model, and use them as fault detectors. Real-acquired data show the ability of the proposed method to detect partial shadowing, open-circuit, short-circuit, and degradation, but the authors only present seven case-studies, which does not demonstrate the generalization of the model for other fault scenarios. Additionally, because the model is based on a decision-tree classifier, it is restricted to the presented 9.6 kWp PV plant.

In terms of dynamic modeling, most of the models are dedicated to energy forecasting and do not present fault detection results. In [16], for instance, a black-box modeling is used to obtain an empirical model for the system, using temperature and irradiance as input signals. However, that paper simplifies the model by excluding possible system nonlinearities, which makes it difficult to use in the context of fault detection. In [36], on the other hand, the Hammerstein–Wiener model is used to emulate the system including nonlinearities. The irradiance and DC power were used as input and output signals, respectively. The chosen sampling time was 15 min., compromising the detection of short-term events, such as partial shadowing. In [37], an ARMAX model is proposed to predict the generated power, one-day ahead, for a PV system. The input signals of the ARMAX model are the daily average temperature, the precipitation, the insulation duration, and the humidity. However, the authors did not discuss fault detection with the proposed model.

It is also noteworthy that none of the discussed detection methods presented the results of the solution in a dedicated hardware or system, integrated to a monitoring system.

### 2.4. Fault Classification

One way to perform fault classification, which has been receiving increasing attention and popularity in recent literature [9], is the use of artificial intelligence models, especially machine learning classifiers, which is also the main approach that is proposed in this work. In [10], for instance, the use of artificial neural networks to classify the operation of a photovoltaic system in four possible states (normal, degradation, short-circuit, and shadowing) is presented. This method was trained and tested in a simulated environment and obtained an accuracy of approximately 88.89% when considering the nine evaluated test samples. Real fault cases were not reported in that work.

In [38], a two-stage system is discussed, being the first for fault detection and the second for classification. The authors consider the following cases: open-circuit, degradation, short-circuit, and shadowing, including or not the bypass diode. For fault detection, the proposed method is based on the comparison of the power of the PV plant with its correspondent mathematical model. When a difference above a given threshold is verified, the system reports a fault detection. For fault classification, a multilayer perceptron artificial neural network is used, reaching an overall accuracy of 90.3% (detection and classification). Furthermore, this system uses only simulated data for training the network and it is tested with a real plant based on the system’s VxI curve. With that, the real-time classification of faults is unfeasible, since the generation of the VxI curve requires the disconnection of the plant to connect the proposed equipment and perform the detection and classification. Following a similar idea, a detection and classification system is presented in [20], obtaining an accuracy of 94.1%. However, the authors also presented tests only in a simulation environment.

In a more recent approach from [39,40], another two stage architectures were used, but this time non-linear auto regressive models (NARX) were developed to estimate the generated power under different environmental conditions. Next, fuzzy inference models compared the estimated value to the sensed power in order to classify the system into one of a given set of fault configurations, which includes combinations of shadowing, short circuit and open circuit, yielding 98.2% accuracy, using 16 use cases.

Still, in the context of intelligent methods in two stages, in [41,42], systems for detecting normal, open-circuit, and different types of short-circuits were proposed. The two-stage approach of the aforementioned works takes place with the use of probabilistic neural networks, for detection and classification of the referred faults. In [41], two simulated tests were carried out to validate the proposed system, achieving a detection and classification accuracy of 82.34% and 98.19%, respectively while [42] achieves 100% accuracy while using real data for training and validation.

More specialized approaches use methods to detect line-to-line faults in several situations. For example, [43] uses a support vector machine trained with simulated data and tested in a real PV plant which achieves up to 94.74% accuracy to detect short-circuit conditions, while [44] uses a Radial Basis Function Neural Network using irradiance and power as its inputs to detect one or modules disconnections from the photovoltaic system. The system attained 98.1%, 97.9%, and 96.5% accuracy when tested in two plants, one with 2.2 kW and other with 4.16 kW when subject to normal operation, shadowing, and overcast conditions. Another Radial Bases Function Network was used in [45] to classify a 1 kW photovoltaic plant into one of 14 cases, including: Normal, short circuit, cell bypass, shading, ground fault, and nine converter/inverter’s component faults. This system was only tested in simulations and achieved 97% test accuracy.

In [11], on the other hand, a method using a Kernel Extreme Learning Machine and data from VxI curves is presented in order to classify PV faults in the following cases: open-circuit, shadowing, short-circuit, and degradation. To evaluate the performance of the system, three case studies were carried out. The first uses only simulation data for training and testing, whilst the second employs only real data acquired in a 1.8 kW peak PV plant. The third approach uses simulated data for training and real data for testing, due to the limited amount of data collected in the real plant. In the case that uses only simulated data, the proposed system reached an accuracy of 100.0%. In the case with real data, the accuracy varied between 97.9% and 99.0% and, in the third case, with mixed data, the final accuracy was 98.9%. Despite the relatively high performance (>95.0%), because the method is based on VxI curves, the PV system must be disconnected to perform the proposed fault classification procedure—it uses an external device that must be connected to the plant to obtain VxI curves. Additionally, the authors did not present the results of the solution in a dedicated hardware or system.

### 2.5. Contributions of This Work

Based on the exposed so far, it can observed that fault detection and classification is a hot topic with very interesting contributions so far. This work complements the current literature presenting different contributions with respect to monitoring of PV systems, detection and classification of faults. In terms of monitoring systems, the related works show that, when the monitoring of plant variables is more comprehensive, there is usually no fault analysis. On the other hand, when fault detection is included (embedded) in the monitoring system, the detected faults are limited to certain types of faults. When both are present, the work is only evaluated using simulations or with lower powered PV deployments. With that, the first contribution of this work is the integration of a fault detection and classification approach with an industrial grade embedded photovoltaic plant monitoring system, allowing for the online identification and classification of different PV faults, besides providing a MS integrated to the plant.

Regarding the fault detection, the use and a very detailed comparison of dynamic models for several fault scenarios is still limited in the literature and it can be highlighted as an additional contribution of this work, particularly for real-acquired data. Finally, from the classification point of view, the use of simulated (and validated) data to train machine learning models with different fault conditions, besides testing with several real-acquired data in real fault scenarios, is also briefly evaluated in the literature and can be highlighted as a relevant contribution. Besides, we also present a detailed comparison among some of the most common machine learning methods, pointing to the most suitable model for online fault classification in PV systems.

Another relevant contribution of our work is the fact that the simulator and the training dataset is publicly available to enable straightforward comparison with newly proposed techniques.

Finally, it is worth mentioning that this work is an extension and combination of previous works [8,17,21] of the authors of this paper, which presented initial and individual results of monitoring system, fault detection, and classification, respectively.

## 3. Proposed Monitoring System

Suppose a photovoltaic system composed of a group S of strings each composed of *N* serially connected photovoltaic modules ${\mathrm{PV}}_{\left\{1,\;\cdots \;,N\right\}}$. When an arbitrary string *s*, (s∈S) is subject to an Irradiance (*G*), it generates a voltage $V_{\mathrm{dc}\;,\;s}$ and a current $I_{\mathrm{dc}\;,\;s}$ (and, consequently, and output power $P_{\mathrm{dc}\;,\;s}=V_{\mathrm{dc},s}\;I_{\mathrm{dc},s}$), which are dependent on the ambient temperature *T*, considered constant for ${\mathrm{PV}}_{j}$, ∀j. An inverter is deployed to convert the energy output of the mentioned S string group into a two-phase ac waveform whose voltage is $V_{\mathrm{ac}}$ and current is $I_{\mathrm{ac}}$. The converted power is injected to the utility grid. A data acquisition system is able to collect all of the aforementioned variables with a minimum sample frequency of 1 Hz. The power output may be influenced by the following system faults: (i) short circuit between an arbitrary number of adjacent ${\mathrm{PV}}_{j,\;\cdots \;,k}$ modules, (ii) open circuit of any module string ∈S, (iii) high resistance connection between any adjacent ${\mathrm{PV}}_{j,k}$ module pair, and (iv) module output mismatch due to partial shadowing. We propose a Fault Detection system which uses the acquired data to detect whether the considered system is operating under one of the proposed faults. When a fault is detected, the fault classification is performed by the appropriated algorithm and the result is informed to the user.

**Figure 1:**
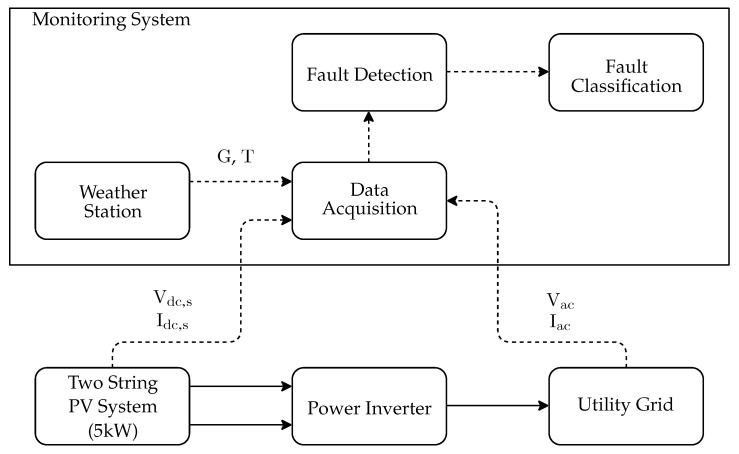
General Overview of the System Model.

**Figure 2:**
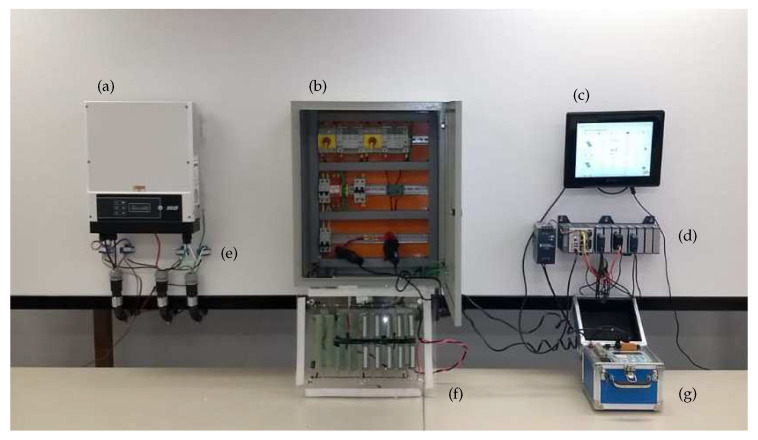
System Inside our Laboratory including: (**a**) Power Inverter, (**b**) Electrical Panel with Protection Devices and Signal Conditioning, (**c**) Local Display, (**d**) CompactRIO and Signal Acquisition Modules, (**e**) alternating current (AC) and direct current (DC) Current Transducers, (**f**) Degradation Resistors, and (**g**) Power Quality Analyzer.

Figure 1 depicts the proposed system in which the acquisition system is implemented by a National Instruments CompactRIO (cRIO) controller equipped with signal acquisition modules, as presented in Figure 2. The cRIO controller runs a Linux Real-Time Operating System and it features a FPGA, and modular I/Os, programmed in the LabVIEW environment, for industrial-grade embedded high-speed control and signal processing systems.

Between the acquired signals and the analysis, there is a software stack that comprises the execution environment where the developed software coordinates the sampling at 25 kHz of $V_{\mathrm{dc},s}$, $I_{\mathrm{dc},s}$, $V_{\mathrm{ac}}$, $I_{\mathrm{ac}}$, with s∈S. Next, the root mean square (RMS) values over the monitored variables are calculated at every second, generating the signals $v_{\mathrm{dc},s}\left(k\right)$, $i_{\mathrm{dc},s}\left(k\right)$,$v_{\mathrm{ac}}\left(k\right)$, $i_{\mathrm{ac}}\left(k\right)$ (with s∈S) which are stored on the local database. Additionally, RMS signals are calculated from the monitored signals: String power output $p_{\mathrm{dc},s}\left(k\right)=v_{\mathrm{dc},s}\left(k\right)\;i_{\mathrm{dc},s}\left(k\right)$ and inverter power output $p_{\mathrm{ac}}\left(k\right)=\;v_{\mathrm{ac}}\left(k\right)\;i_{\mathrm{ac}}\left(k\right)$. At the top of the stack, the fault detection and classification algorithms are implemented. In the following subsections, the blocks from Figure 1 are presented.

### 3.1. PV Power Plant

In this work, ${\mathrm{PV}}_{\left\{1,\;\cdots \;,N\right\}}$ are implemented using N=16, Canadian 330W Poly-crystalline Modules (CS6U-330P), forming a group S, with |S|=2 strings of 8 modules each (where |·| represents group cardinality). Table 1 presents the main electrical data of the module.

**Figure 3:**
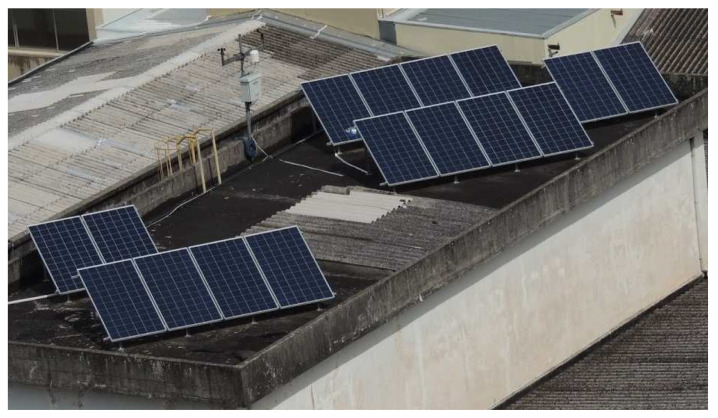
View of PV plant located at −25.438686 (Latitude) and −49.268487 (Longitude)–City of Curitiba–State of Paraná–Brazil.

In Figure 3, the deployment site of the proposed system (modules and sensors) is presented. In total, the system may yield 5 kW peak installed capacity.

Connected to the grid, the power inverter produced by NHS (depicted in Figure 2) is responsible for converting the DC input energy coming from strings 1 and 2 to the single phase AC output. As well as running the Maximum Power Point Tracking (MPPT) algorithm for maximum energy conversion, this inverter can also measure $V_{\mathrm{dc},s}$, $I_{\mathrm{dc},s}$, $V_{\mathrm{ac}}$, and $I_{\mathrm{ac}}$, with s∈S and report its RMS values via a RS-485 interface to the acquisition system. This report may be used to detect external sensor faults.

### 3.2. Weather Station

Because photovoltaic energy production is strongly dependent on the instantaneous environmental conditions where the solar panels operate, it is important for the acquisition system to correlate the instantaneous power output with meteorological variables. For this reason, a weather station was connected to cRIO to monitoring the variables that have the major influence in photovoltaic production: (i) irradiance *G* [W/m${}^{2}$]; and, (ii) module temperature *T* [${}^{{}^{\circ}}$C]. Additionally, our system measures secondary variables that may influence the main variables: (iii) ambient temperature: Ta [${}^{{}^{\circ}}$C]; (iv) relative air humidity: *H* [%]; (v) dew point [${}^{{}^{\circ}}$C]; (vi) wind speed: $W_{s}$ [m/s]; and, (vii) wind direction: $W_{d}$ [degrees]. In Figure 3, the weather station may be observed adjacent to PV modules and sensors.

For the irradiance (*G*) measurement, a EKO MS-40 class B pyranometer was installed adjacent to ${\mathrm{PV}}_{1}$ following the same inclination of the panel installation [19]. It is capable of measuring global irradiance (285 to 3000 nm spectral sensitivity) with 180${}^{{}^{\circ}}$ angle. Panel temperature from ${\mathrm{PV}}_{\left\{1,\;\cdots \;,N\right\}}$ is assessed by four PT100 contact sensors, Kimo Instruments SFCSD-51-A-3-PVC-25, installed on the back side of ${\mathrm{PV}}_{1,6,11,16}$: *T* is considered to be the arithmetic average of the obtained measurements. The output accuracy of these sensors for temperatures between 0 and 100 ${}^{{}^{\circ}}$C is ±0.15 ${}^{{}^{\circ}}$C. The environmental signals are obtained from a Novus Fieldlogger datalogger (eight analog input channels, with 24 bit A/D resolution and 1 kHz maximum sampling rate). The connection to the cRIO is implemented through Modbus TCP/IP bus over ethernet. The datalogger is factory calibrated, so no further processing is necessary on the main monitoring system, besides synchronization and logging. The datalogger reports the 1 s average of *G* and *T* to the acquisition system, which are respectively named g(k) and t(k) in the following sections.

### 3.3. Electrical Variables

**Figure 4:**
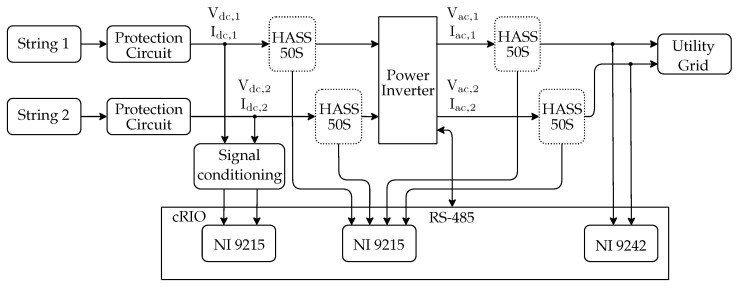
Diagram showing the electrical variables monitoring system using the cRIO RS485 interface to the power inverter, two NI 9215 and one NI9492 acquisition cards.

Figure 4 shows a detailed view of the electrical variables acquisition scheme. There, the exact points of acquisition of monitored signals are indicated for DC voltage and current output for both PV strings ($V_{\mathrm{dc},s}$, $I_{\mathrm{dc},s}$ with s∈S), as well as the AC voltage at the output of the power inverter ($V_{\mathrm{ac}}$, $I_{\mathrm{ac}}$), which are obtained by measuring both wires of the inverter with respect to the Utility Grid neutral wire (not shown for simplicity reasons) ($V_{\mathrm{ac},1}$, $I_{\mathrm{ac},1}$, $V_{\mathrm{ac},2}$, and $I_{\mathrm{ac},2}$).

One NI 9215 module is used to acquire DC voltage signals of both strings. It reads two simultaneously sampled analog input channels. Besides surge protection already installed in the power plant, a signal conditioning and protection circuit was installed in order to adapt the range and protect the acquisition cards for DC voltage of the PV strings measurements.

The current transducer used is the LEM model HASS-50S. It is based on the Hall effect measuring principle with frequency bandwidth up to 50 kHz, and galvanic isolation between primary and secondary circuit. All of the current signals, DC and AC, are acquired by a second NI9125 card.

For AC voltage signals, an NI 9242 module is used. It offers three channels for measurements between the signal and the neutral channel, and the neutral channel provides measurements between its terminal and the chassis ground.

Regarding the measurement of electrical power, both in DC and AC domains, they are calculated in real-time using the sampled voltage and current, as advised by EN 61724:1998 standard [25]. Additionally, phase dependent quantities in a two-phase unbalanced power system (which is our scenario), such as power factor (PF), total and apparent power flow, and total harmonic distortion (THD), were implemented in the frequency domain and validated in accordance with IEEE1459 [46].

The measurements of voltage and current DC and AC, as well as aggregated values, such as active and reactive power, including the signal conditioning, were calibrated against local instruments, namely a Tektronix model MDO3014 oscilloscope and a RMS model MarH21 Power Quality Analyser (presented in Figure 2). The resulting accuracy was inferior than 1% for all signals, as recommended in EN 61724:1998 standard [25].

### 3.4. Execution Environment and Developed Software

In general, the MS reads environmental variables, acquires, calibrates, and aggregates electrical signals, time stamps the data, and provides a database driven execution environment where Fault Detection and Fault Classification can be implemented.

**Figure 5:**
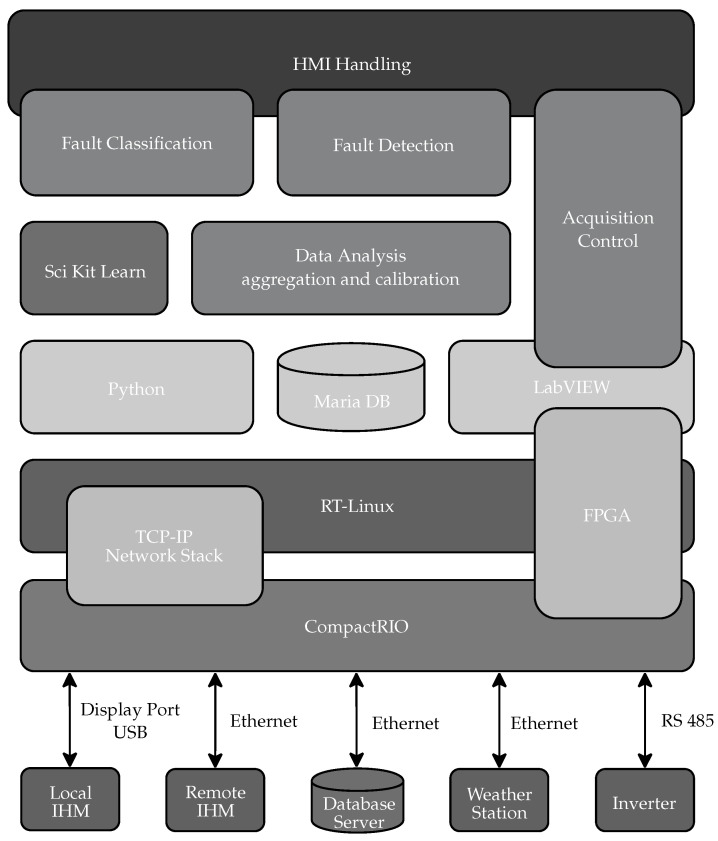
Software stack of the monitoring system, including the applications at the toplevel and hardware and interfaces at the bottom level.

Figure 5 shows the software stack, including the bottom level hardware modules, which implement the interfaces to the real-world, already described in the previous section. The Acquisition Control module coordinates the cyclical process from reading to storing. The LabVIEW synthesized programmable logic running in FPGA guarantees the real-time execution and synchronization of the sampled signals at 25 kHz. The RS485 Modbus communication with the power inverter, and the Ethernet connection to the weather station, are implemented as sub-Virtual Instruments (function) in LabVIEW environment and run at 1s period.

The Data Analysis module is responsible for calibration and aggregated values calculations, prior to the storage in the database, with timestamps via the Ethernet connection as well. Another functionality implemented in the Data Analysis module is the interpolation, decimation of timestamped signals and aggregated values, and its comparison in order to update the Human Machine Interface (HMI), generating events for user configurable alarms. It runs as a software in Python over a TCP-IP remote procedure call. This scheme allows doe the higher level applications to run locally in the cRIO, as well as in another network node of a distributed system.

The logical connection to the database server running Ubuntu is done through a Maria DB client installed directly in the Linux environment in the cRIO, and activated through shell scripts from LabVIEW and Python. The database server in the local network can be expanded to the cloud as a scalable solution for multiple power plants management.

At the top of the software stack, the higher level applications are the Fault Detection module, running over LabVIEW, and the Fault Classification module, running in Python. The classification module uses the SciKit Learn machine learning libraries as middleware.

Finally, the HMI Handling module, implemented in LabVIEW, coordinates the system graphical user interface, presented in a touch screen display directly connected to the cRIO USB and Display Port connections. Using this HMI, it is possible to visualize real time data and generate a graphical analysis for configurable time windows, as well as setup alarm conditions to be indicated by the system.

## 4. Datasets

The proposed fault detection and classification systems are based on system identification and machine learning techniques, which may require a large dataset of past operational data for training purposes, particularly for machine learning models. The accuracy of these algorithms in the different situations relies on the diversity of this training dataset, which must contain operational data under all of the considered faults for the whole range of environmental conditions.

We created a PV plant simulator that can generate the required dataset in a short period of time since waiting for the natural occurrence of all these environmental combinations to generate the required faults is impractical for most PV installations. On the other hand, the generated model must accurately describe a real system behavior. This way, we use the real photovoltaic installation (Section 3.1) to validate our simulator setup. This hybrid approach used to generate our training and validation dataset will be described in the following sections. First, the methodology to artificially introduce operational faults in the real installation is described. In the sequence, the proposed electrical simulator that matches the real installation behavior is presented.

### 4.1. Real System Installation

**Figure 6:**
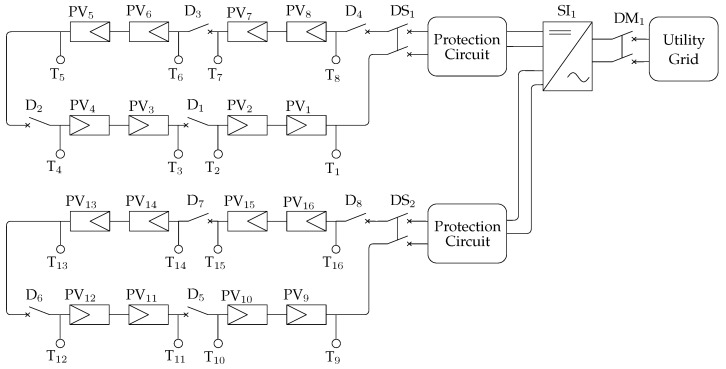
Installed Photovoltaic Plant Electrical Diagram.

Figure 6 details the electrical diagram of the installed PV plant of Figure 3, where 16 photovoltaic modules (${\mathrm{PV}}_{\left\{1,\;\cdots \;,16\right\}}$) are arranged in a group S of two eight-module photovoltaic strings. The string outputs are connected to a 5 kW grid tied inverter (${\mathrm{SI}}_{1}$). Still in that same installation, an electrical panel houses auxiliary components that are usual in a photovoltaic installation: Fuses and Transient Voltage Supressors (Circuit Protection Block), String Circuit Breakers (${\mathrm{DS}}_{\left\{1,2\right\}}$), and Main Circuit Breaker (${\mathrm{DM}}_{1}$). Additionally, some components are installed to generate system faults: twelve sockets $T_{\left\{1,\;\cdots \;,12\right\}}$ may be connected to generate diverse short-circuit conditions or be used in conjunction with auxiliary circuit breakers ($D_{\left\{1,\;\cdots \;,8\right\}}$) to insert arbitrary resistances (Degradation Resistors in Figure 2) in series with the strings, generating degradation faults. The same circuit breakers ($D_{\left\{1,\;\cdots \;,8\right\}}$) may also be used individually to generate open-circuit faults. Finally, shadowing may be generated by physically blocking solar radiation using diverse opaque objects.

We start from completely clear days to generate the real dataset: cloudy and rainy days are not considered, since there is no way to guarantee that natural shadowing is not influencing the collected parameters. Next, the fault schedule that is presented in Table 2 is generated in the system.

Partial shadowing occurs naturally due to the characteristics of the deployment site: sunlight is blocked by a nearby buildings in different moments of the morning and afternoon (around two hours in each period). The process was repeated for 16 days, when data is collected and properly labeled from around 07:30 to 17:00, including faults and normal conditions, with a sampling ratio of 1 Hz, generating 10,371 points with degradation, 5999 points with short-circuit, 6024 points with open-circuit, 184,311 points with partial shadowing, and 309,253 points in which no faults were introduced. This real dataset is also publicly available (https://github.com/clayton-h-costa/pv_fault_dataset) in order to facilitate other experiments regarding fault detection and classification methods.

### 4.2. System Simulation

One major concern taken into consideration, when our PV simulator was developed, is the ability to represent different commercially available components used in PV plants. For validation purposes, the parameters that are chosen for Dataset generation match the ones from the available system described in Section 3.

**Figure 7:**
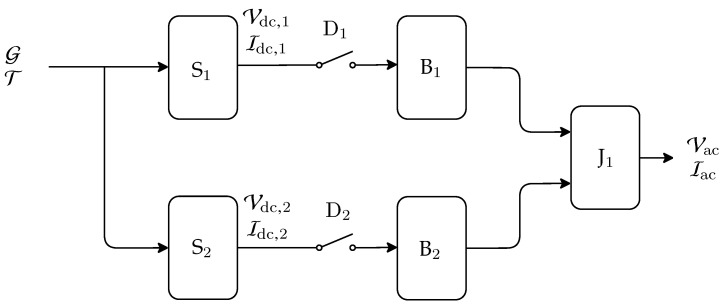
Photovoltaic Simulator System Architecture.

The electrical circuits of simulator runs on PSIM (Power System Simulation software), while the environmental variables are controlled by a Simulink/Matlab script. The implemented simulation blocks can be observed in Figure 7, where $S_{\left\{1,2\right\}}$ models PV strings configured with eight PV modules with 330 W each, which are individually simulated using the single-diode model [33], which takes the cell’s irradiance and temperature as inputs. The parameters of the model were chosen to match the PV panels specification from Table 1, as detailed in [21]. Next, G represents the simulated system irradiance, while T represents the simulated module temperature.

Each $S_{s}$ module outputs a simulated voltage $V_{\mathrm{dc}},s$ and a simulated current $I_{\mathrm{dc}},s$, which are inputs for a fixed voltage output boost converter ($B_{s}$), which implements a perturb and observe MPPT algorithm [47] (with s∈{1,2}). Finally, the regulated output is fed into a full bridge inverter ($J_{1}$) that converts the DC bus to a 127 V, 60 Hz, single phase output connected to a simulated utility grid.

Additionally, some auxiliary elements were introduced to simulate the considered system faults: switches that model open circuit faults, resistors that model string degradation, a variable that models partial shadowing, and switches that model short-circuit faults. Details of these elements can also be observed in [21].

Training dataset was generated using the simulation by covering each of the five operational conditions for the whole range of temperature (T) and irradiance (G). The lower temperature bound was set to −5 ${}^{{}^{\circ}}$C, which is compatible to historical minimum for the installation site (Curitiba-Brazil), while the upper bound was set to +85 ${}^{{}^{\circ}}$C which is the maximum operational temperature of the PV module. The irradiance range was set from 100 W/m${}^{2}$ to 1000 W/m${}^{2}$ which represents a range between the point at which the real inverter starts to operate and the peak generated power. The temperature range was simulated in 19 steps with 5 ${}^{{}^{\circ}}$C each while the irradiance was simulated in 19 steps of 50 W/m${}^{2}$ for each of the four considered faults. For the shadowing fault, four different cases were simulated, each with a set amount of shade varying from 5% to 15% of a string, which is the typical shadowing observed in the PV plant. This setup resulted in 361 sample cases per string, with 5054 samples in total.

## 5. Fault Detection

Fault detection technique, as expressed in this section, is based on modelling the photovoltaic system dynamics, and uses the matching between the real system and the model as a metric of properly operation of the system. In this context, it is important to clarify two concepts as: (i) system: defined as confined arrangement of mutually affected entities [48]; and, (ii) model: defined as mathematical representation of these systems [49]. The next section will bring more details about the PV modeling process.

### 5.1. System Identification Model

In this work, the methodology that was used to achieve proper models for real systems was based on system identification. In this context, one can define system identification as a method of measuring the mathematical description of a system by processing the observed inputs and outputs of the system [50]. Generally, a model achieved by this technique is more accurate to describe a system than models based only on physical laws [48]. In the scenario of this work, it is possible to apply models in order to detect miss-functions of an specific system [51].

It is important to mention that only the real dataset (detailed in Section 4.1) was employed in order to estimate the detection model parameters.

### 5.2. Proposed Fault Detection Method

**Figure 8:**
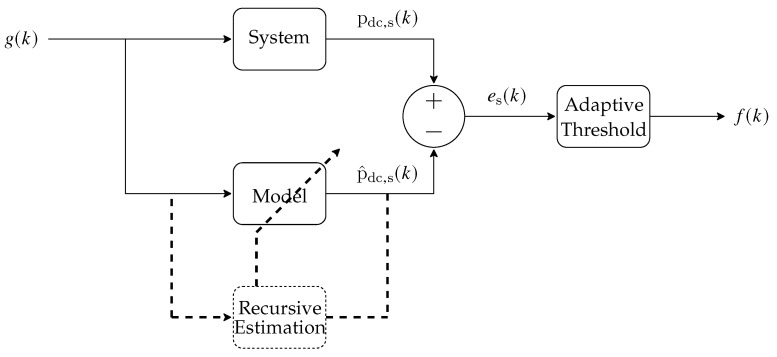
Example of an output error model, with instantaneous irradiance g(k) as an input, an instant power $p_{\mathrm{dc},s}\left(k\right)$ as output, an estimated output ${\hat{p}}_{\mathrm{dc},s}\left(k\right)$, an error e(k), and a fault f(k).

The idea behind using dynamic models to detect faults is based on the mismatching between the output of the real system, instant power $p_{\mathrm{dc},s}\left(k\right),s\in S$, and the model’s output, ${\hat{p}}_{\mathrm{dc},s}\left(k\right)$, its estimation. More specifically, in a photovoltaic array, the model only represents the system’s output, if this system is operating without miss-functions. If there is some fault, then the model and PV plant will operate with different dynamics. Consequently, the residue $e_{s}\left(k\right)$, given by $e_{s}\left(k\right)=p_{\mathrm{dc},s}\left(k\right)-{\hat{p}}_{\mathrm{dc},s}\left(k\right)$, will increase as time goes by. If the signal $e_{s}\left(k\right)$ reaches an amplitude greater than a limit established by the Adaptive Threshold block, so one can conclude that there is a fault, f(k), in the power plant. Figure 8 shows the general idea of this process.

**Figure 9:**
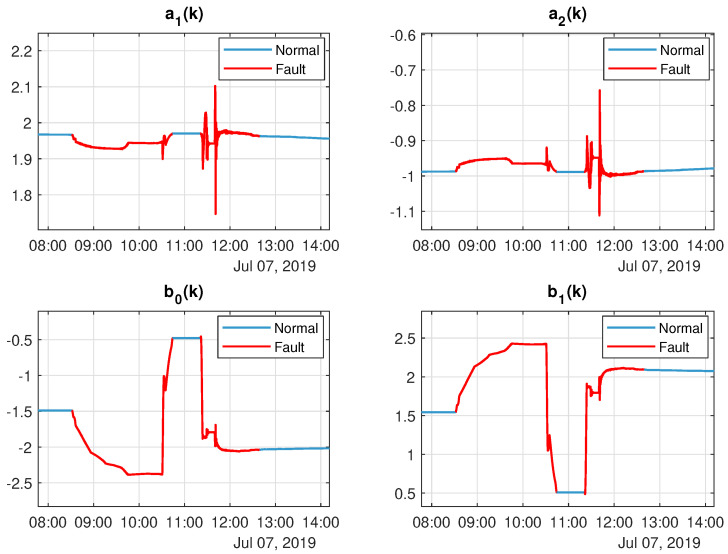
Model parameter evolution over time. The sample period employed was 1s and the parameters considered are: $\theta ={\left[a_{1}\left(k\right)\;a_{2}\left(k\right)\;b_{0}\left(k\right)\;b_{1}\left(k\right)\right]}^{T}$. The fault instants are highlighted in the figure.

In this work, the dynamics of the underlying system was approximated by a linear Auto-Regressive with eXogenous input (ARX) model, expressed by

(1) $${\hat{p}}_{\mathrm{dc},s}\left(k\right)=a_{1}\;{\hat{p}}_{\mathrm{dc},s}\left(k-1\right)+a_{2}\;{\hat{p}}_{\mathrm{dc},s}\left(k-2\right)+b_{0}\;g\left(k\right)+b_{1}\;g\left(k-1\right),$$

or one can obtain the system’s transfer function, using *z* transform

(2) $$\frac{P(z)}{G(z)}=\frac{b_{0}+b_{1}\;z^{-1}}{1-a_{1}\;z^{-1}-a_{2}\;z^{-2}},$$

in which G(z)∈C is the *Z* Transform of the irradiance, g(k), and P(Z)∈C is the *Z* transform of the output power, ${\hat{p}}_{\mathrm{dc},s}\left(k\right)$, both in frequency domain. Parameters’ vector $\theta ={\left[a_{1}\left(k\right)\;a_{2}\left(k\right)\;b_{0}\left(k\right)\;b_{1}\left(k\right)\right]}^{T}\in R^{4}$ were estimated using a Recursive Least Squares (RLS) algorithm [52]. As the system can have its dynamic varing over time (after a fault or degradation process), the evolution of these parameters can be seen in Figure 9.

It is worth mentioning that there are two strings in the underlying photovoltaic system. Thus, in order to detect faults in each string, there are two models as shown in Figure 8 (one for each string). Additionally, in each model, the parameters $\theta ={\left[a_{1}\left(k\right)\;a_{2}\left(k\right)\;b_{0}\left(k\right)\;b_{1}\left(k\right)\right]}^{T}$ are approximately constant in normal system’s operation. The discontinuities presented in the Figure 9 (dashed red lines) are representing possible faults in the photovoltaic system.

Table 3 shows the correlations that were calculated between many possible input variables and the power output, in each string, of the underlying system, using real data, collected from the PV system and the meteorological station. Taking that into account, one can see for both input variables, irradiance (*G*) and PV temperature (*T*), a high correlation with output power in String 1 ($P_{\mathrm{dc},1}$), and the same for output power in String 2 ($P_{\mathrm{dc},2}$). Therefore, the natural choice for input variables would be: (i) irradiance (*T*); and, (ii) PV temperature (*T*). However, for simplicity reasons, only the irradiance *G* was chosen as input variable, because of its higher correlation: 0.96.

Furthermore, the linear ARX model was chosen because of the following reasons:

- ARX linear models are one of the most simple dynamic systems. Thus, its computational implementation is less time consuming, which is ideal for the implementation of monitoring systems in photovoltaic area;
- The ARX model represents a close approximation to the real system, as detailed in Section 7.

It is important to mention that the proposed model is not the only one. In a previous work, the authors have already investigated Diode Models and Hammerstein-Wiener models, applied to the same problem [17]. The comparisons will be presented in Section 7.

In order to detect faults using the mismatch between the system and model, an adaptive threshold was employed, as can be seen in Figure 8. This block was mathematically composed by a recursive mean, expressed in (3), and recursive variance, expressed in (4), both being modulated by a forgetting factor λ [51]:

(3) $$\bar{e}\left(k\right)=\lambda \;\bar{e}\left(k-1\right)+\left(1-\lambda \right)e\left(k\right),$$

(4) $$\sigma^{2}\left(k\right)=\frac{2\;\lambda -1}{\lambda }\;\sigma^{2}\left(k-1\right)+\left(1-\lambda \right)\left[e\left(k\right)-\bar{e}\left(k\right)\right],$$

in which $\bar{e}\left(k\right)\in R$ is the mean of residues, in instant k∈Z, λ∈[0,1], and $\sigma^{2}\left(k\right)\in R$ is the variance of the residues. One event is considered as a fault if the absolute value of the residue, |e(k)|, overflows the threshold $\bar{e}\left(k\right)\pm ⋉\;\sqrt{\sigma^{2}\left(k\right)}$, i.e.:

(5) $$\left\{\begin{array}{c} \mathrm{if}\;|e\left(k\right)|\;>\;max\left(|\bar{e}\left(k\right)|\pm ⋉\;\sqrt{\sigma^{2}\left(k\right)}\right),\;f\left(k\right)=1\;\left(\mathrm{fault}\right);\\ \mathrm{otherwise},\;f(k)=0\;(\mathrm{normal}\;\mathrm{operation}); \end{array}\right.$$

The selection of the value ⋉ was empirically done, aiming at minimizing Mean Square Error (MSE) between f(k) and a benchmark signal, $b_{f}\left(k\right)\in B=\left\{0,1\right\}$, expressing the evolution of manipulated faults over time. In other words, $b_{f}\left(k\right)=0$ defines that there is no fault in the system, while $b_{f}\left(k\right)=1$ represents a manipulated fail occurring.

### 5.3. Detection Metrics

In order to quantify the results that were obtained by the fault detection process, some metrics were employed in this work. However, before defining performance metrics for this process, it is important to define the following concepts:

- TP (True Positive): it happens when the detection process points out a real fault, in the photovoltaic system;
- TN (True Negative): it occurs when there is no fault in the photovoltaic system, and the fault detection system confirms that;
- FP (False Positive): it happens when the photovoltaic system presents no fault, and the fault detection system points out a fault; and,
- FN (False Negative): it occurs when the photovoltaic system presents a fault and the detection system does not signalize it.

Based on this, one can define the following performance metrics for the proposed fault detection system:

- Accuracy (A): corresponds to the overall detection efficiency:
  (6) $$A=\frac{TP+TN}{TP+TN+FP+FN};$$
- Precision (P): stands for the rate between positives indicators:
  (7) $$P=\frac{TP}{TP+FP};$$
- Sensitivity (S): evaluates the efficiency of classifying correct detection:
  (8) $$S=\frac{TP}{TP+FN};$$
- Specificity (E): evaluates how efficiently the classifier identifies incorrect detections:
  (9) $$E=\frac{TN}{TN+FP}.$$

## 6. Fault Classification

Whenever a fault is detected at a given time (f(k)=1), the fault classification block is responsible for indicating to the user the most probable cause of the abnormal operation. For this task, we evaluated the accuracy of the four most common supervised machine learning methods. The variables that were chosen as the input for these algorithms are the ones that describe the behavior of the DC side of the PV plant, where the faults occur, forming a feature vector

(10) $$\mathrm{FV}\left(k\right)=\left[\begin{array}{cccccc} g(k) & t(k) & v_{\mathrm{dc},1}\left(k\right) & v_{\mathrm{dc},2}\left(k\right) & i_{\mathrm{dc},1}\left(k\right) & i_{\mathrm{dc},2}\left(k\right) \end{array}\right].$$

The classification system may be represented as a mapping function h:FV(k)→Ψ, where $\Psi =\left\{L_{\mathrm{short}},L_{\mathrm{open}},L_{\mathrm{degradation}},L_{\mathrm{shadowing}}\right\}$ represents a group of the four considered faults. Every considered method uses a training procedure to construct *h*. A brief description of each method and the training procedure will be detailed in the next paragraphs.

The first method tested was k-Nearest Neighbors [53], in which the current feature vector of the PV plant is associated to the one in the training set which presents the most similar characteristics (closest in terms of Euclidean distance). This method is very simple to implement, but has an disadvantage of requiring the complete training set to perform the classification.

For this reason, a still simple but less memory intensive method was considered: Decision Trees [53]. In this method, a tree structure is formed in which each class is contained in a leaf node. For classification, this tree is traversed from node to leaves, with each step being guided by binary decisions based on different input features. As an advantage, the classification procedure is very simple, but the process of building an efficient unbiased decision tree is not always available. Furthermore, a slight change on classes may require a complete tree rebuild, which motivates the search for more efficient classification algorithms.

Another widely used classification method is Support Vector Machines [54], which operates by creating a multidimensional vector space in which each feature vector is represented as a point. The training procedure consists in determining an hyperplane mapped so that the examples of the separate classes are divided by a margin that must be made as wide as possible. The solution for the problem can be found with convex optimization techniques.

The last compared method was artificial neural networks, which consists of a parallel distributed signal processor that have the capacity to store knowledge using a learning algorithm. In this work, we used the Multi-Layer Perceptron (MLP) network [53], which is a feedforward architecture, with just a single hidden layer, an input layer, and the output layer, which corresponds to the assigned class (label). The learning process is based on the backpropagation algorithm [53], which basically consists in a method to estimate the gradient of the training error cost function, along the layers of the network, allowing the use of a gradient descent-based method to optimize and estimate the parameters. All of the mentioned algorithms were trained using the procedure depicted in the next section.

### 6.1. Training Procedure

**Figure 10:**
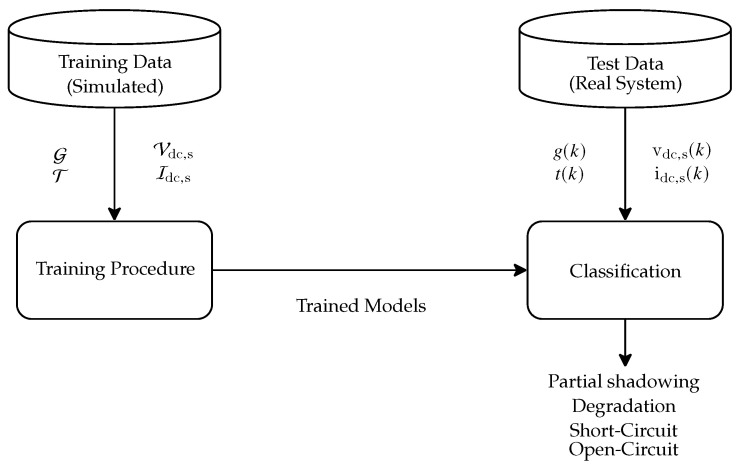
Training and Test Procedures for Fault Classification.

The training and test procedures for fault classification are depicted in Figure 10. First, the training data, composed of only simulated data generated with the parameters presented in Section 4.2, are used to train the supervised machine learning methods, generating the mapping function *h*. Subsequently, *h* is applied to labeled points of the real dataset, as presented in Section 4.1. The partitioning generated by the mapping function is then compared to the actual label partitioning, this way, the system accuracy can be determined.

In the training procedure, a technique, called cross-validation [53], was used with the train data to set the parameters for the classification methods. In that technique, the training dataset is partitioned into two groups, the first one is used for training while the second is used for classification accuracy evaluation (Section 6.2. The process can be repeated several times, by using different data to compose each partition, attention must be paid to include a fair proportion of each classification labels on the training/validation groups. The final result is the average of all repetitions.

In our case, we partitioned the simulated dataset using 80% of data for training, and repeating the process 10 times by using a different data to compose the partitions. This cross validation process was run with varying values for the method’s parameters, in which we chose the ones that yield the best accuracy rate average among the tests.

For k-Nearest Neighbors, the parameter k, which is the amount of nearest neighbors considered, was varied in the set {2,3,⋯,99,100} for the cross-validation application. Regarding Decision Tree, two parameters were evaluated: Maximum Leaf Nodes and Maximum Depth. The boundaries of the variation were {2,3,⋯,99,100} for both parameters. For Support Vector Machine, the best results were achieved using the Gaussian kernel [54], which contains two parameters to be varied in the cross-validation process: the regularization parameter *C* (searched in {0.5,1,2,5,10,15,20,25}), which is responsible for the balance between model complexity and goodness of fit to the training data and the kernel parameter γ (searched in {0.1,0.5,1,2,5,10,15,20,25}). Finally, the ANN was built with six input units, four output units and a single hidden layer with its size as the sole hyperparameter used. The hidden layer uses the Rectified Linear Unit as activation function, while the output layer uses the softmax (normalized exponential function). Additionally, the optimization solver used was the Adaptive Moment Estimation, or ADAM, as presented in [55]. The search for the hidden layer size, via cross-validation, was made in the interval {5,6,⋯,29,30} neurons.

### 6.2. Classification Metric

For overall performance assessment of the classification stage, one can construct a confusion matrix with *J* classes, being *J* equals to the number of faults. Using this confusion matrix, the performance for all classes in terms of classification accuracy can be calculated, while using individual performances per class. The individual performance is computed by the number of corrected classified examples divided by the total number of examples per class. Subsequently, the average of individual performances is used to reduce the impact of class imbalance on the final result, allowing for a more adequate comparison among all methods.

## 7. Results and Discussion

In this section, all of the results are presented and discussed. First, the results of individual fault detection are presented, comparing recursive approaches and indicating the most appropriate model proposed in this work. In the sequence, the validation of simulated data for classification is presented, in order to highlight the use of simulated data in the context of classification. Posteriorly, the results of individual fault classification are presented, for different machine learning models. Subsequently, a combination of the best fault detection and classification models is presented, followed by a comparison with state-of-the-art models. Finally, the results of the MS with integrated fault detection and classification are depicted.

### 7.1. Fault Detection

#### 7.1.1. Model Results

**Figure 11:**
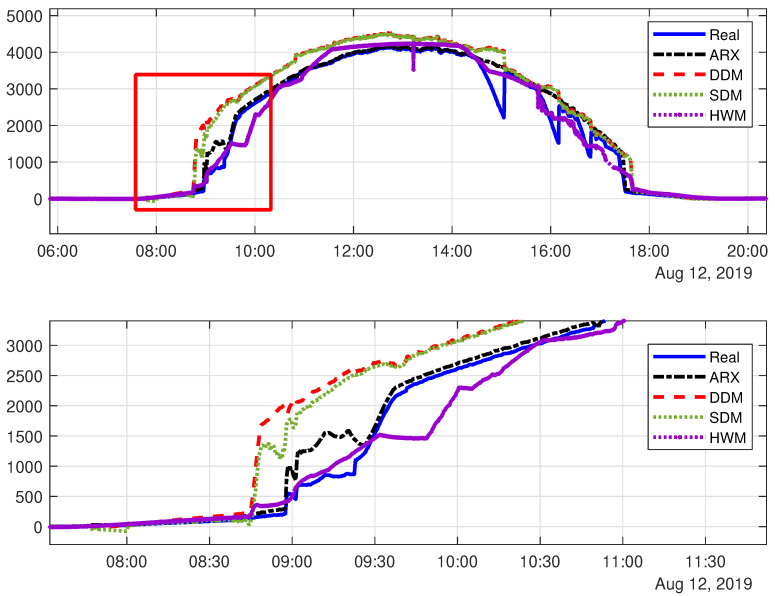
General comparison (top) between real output system and Single-Diode Model, Double-Diode Model, and Auto-Regressive with eXogenous input (ARX) model, with a detailed view between 08:30 and 10:00 in the bottom.

This section presents the results of the fault detection process based on photovoltaic models and adaptive thresholds. Figure 11 shows, graphically, the performance of Single-Diode Model (SDM), Double-Diode Model (DDM), ARX model, and Hammerstein–Wiener Model (HWM) as compared to the real output system.

Table 4 presents a quantitative comparison, between the different possible models. This analysis is based on Normalized Root Mean Square Error (NRMSE).

**Figure 12:**
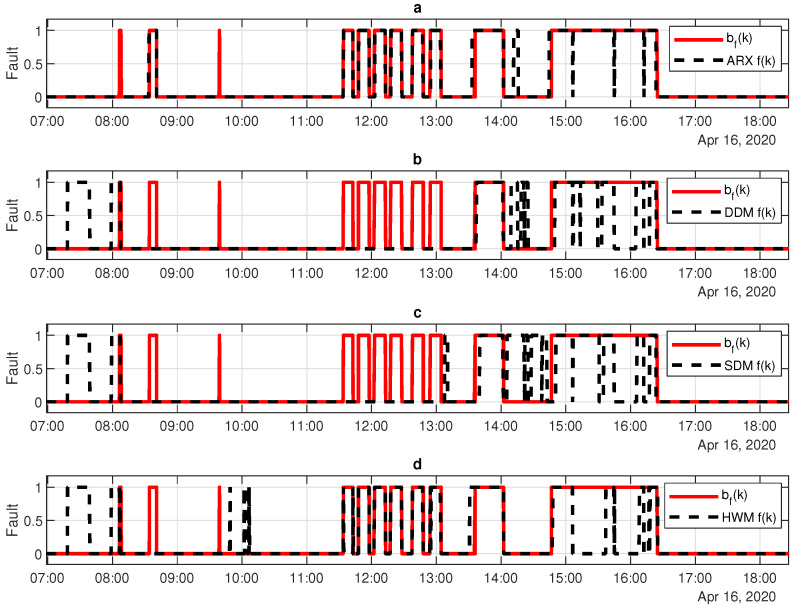
Example of a detection fault f(k), compared with the manipulated fail $b_{f}\left(k\right)$, for: (**a**) ARX model; (**b**) DDM; (**c**) SDM; and, (**d**) Hammerstein-Wiener model.

Based on the Models NRMSE values, Table 4, and on Figure 12, it is possible to conclude that ARX model overcame the Diode models, reaching a NRMSE of 0.82, (for more details about the DDM, refer to [56]). However, the Hammerstein–Wiener (for more information, see [17]) has reached a NRMSE of 0.84. It is worth mentioning that, in this part of the analysis, only data representing the system without fault was employed on the parameter estimation of the models. It was expected that the DDM surpassed the SDM, in terms of NRMSE. However, in these experiments using real data, the SDM achieved a NRMSE bigger than DDM, 0.77 against 0.76. The authors consider that it happened because of the sub-optimal parameters estimation employed in DDM.

Analyzing the reasons for the ARX model performance, it may occur: (i) because the ARX model is dynamic, by definition, while the Diode models are static, and have difficulties for representing transient events in the underlying system; and, (ii) the employed ARX model parameters’ estimation is based on RLS algorithm, which is better adapted to model a time varying system, or a system in fault operation.

#### 7.1.2. Fault Detection Results

This section describe the results of applying the models, as mentioned in Section 7.1.1, in order to detect faults in photovoltaic systems. Figure 12 shows an overlay of the fault benchmark function $b_{f}\left(k\right)$ and fault, f(k), based on: (a) ARX model; (b) DDM; (c) SDM; and, (d) Hammerstein–Wiener model, all of them followed by an adaptive threshold.

The results obtained from fault detection process, based on SDM, DDM, ARX model, and HWM, are presented in the Table 5, Table 6, Table 7 and Table 8, respectively.

Table 5, Table 6, Table 7 and Table 8 show the raw data used to calculate the overall efficiency of the proposed fault detection process (Table 9). However, some inferences can be done even using the raw data. Among the tested cases, ARX models (Table 7) present: (i) the best ability to point out correctly when the system is operating normally (298,641 detections); (ii) the best capacity to discern when the system is operating in fault (181,658 detections); (iii) the lowest number of false positives (10,612 detections); and, (iv) the lowest number of false negatives (25,047 detections). These characteristics and the performance comparison among the models can be summarized in the Table 9.

Based on Table 9, one can conclude that ARX model approach overcomes the three other models in all analyzed statistical properties. It is important to point out that the overall accuracy offered by the ARX model is 19.64% greater than the second best model (DDM). It is also worth to mention that the detection fault (based on ARX model) is robust in signalizing that the photovoltaic system is not in fault state, presenting a specificity of 92.26%. On the other hand, its precision is under 90%, representing the detection system can indicate faults erroneously in 12.22% of cases (considering a precision of 87.88%).

It is important to mention that the HWM was tested in fault detection, in spite of its time invariant nature, which is not advisable for detecting a fault, and its higher computational cost (if compared with the others). The SDM and DDM were tested, because they are benchmarks largely employed in the literature (as presented in Section 2.3 and Section 4.2). Furthermore, all of the mentioned models were coupled with an adaptive threshold, in order to carry out the fault detection process.

The better performance achieved by the ARX model in fault detection may be due to: (i) it is a dynamical model (and have advantages if compared with static diode models); (ii) as ARX models are dynamic, they can describe the system behavior in both transient and steady state; and, (iii) ARX model parameters estimation is based on RLS algorithm, which is better adapted to model a time varying system (surpassing time invariant models, as Hammerstein–Wiener, in a fault detection).

### 7.2. Validation of Simulated Data

In Section 4, we proposed a hybrid approach for generating the training dataset. Some preliminary tests were made in order to guarantee that the simulated systems accurately emulates the real system behavior. For these tests, all of the collected irradiance (g(k)) and temperature (t(k)) pairs from the real dataset (Section 4.1) were used to generate simulated outputs.

**Figure 13:**
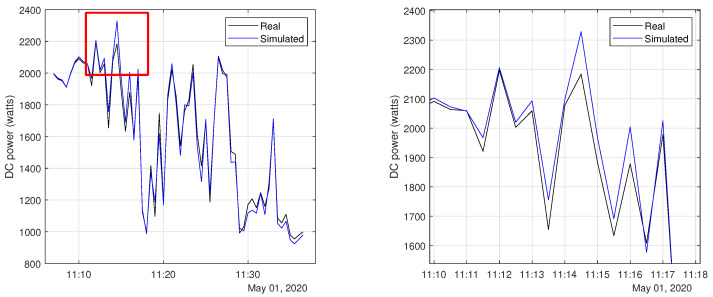
Comparison between simulated and real DC power output when the same irradiance (*G*) and temperature (*T*) pairs are used.

Table 10 shows the Mean Absolute Percentage Error (MAPE) between real and simulated data for normal operation and for each of proposed faults except from open-circuit, which outputs zero. It can be seen from the results that a maximum MAPE of 2.59% is observed for the normal operation, which is sufficiently small to not affect classification accuracy, when the system is trained using simulated data only. In addition, an excerpt of the generated data was plot in Figure 13, which shows a visual concordance between real and simulated data.

Because the validity of the proposed simulator is considered to be sufficient, we can proceed to Fault Classification tests, which will be described in the next section.

### 7.3. Fault Classification

We started the assessment of the classification methods by determining which algorithm is the best suited in this stage. Table 11 shows the obtained results, as well as the optimal parameters that are found with the cross-validation method, from Section 6.1. The Table also shows two accuracies: the Train Accuracy was calculated by the classification of the 20% data reserved for validation on the training dataset, whereas the Test Accuracy was calculated using the classification of the real dataset.

It can be observed that Decision Tree is the least accurate of the classifiers for our use case and the one that presents the most severe performance degradation when applied to real data. Surprisingly, SVM and k-NN classifiers performed better with real data than with the validation data. Such a result may be justified by the fact that, in the training set, there are extreme temperature values that are not observed in real data. Those conditions resulted in poor classification results in the training set, but are not included in the test set, since temperature is not less than five ${}^{{}^{\circ}}$C and not superior than 32 ${}^{{}^{\circ}}$C for real data. Additionally, SVM and k-NN classifiers cannot perform better than artificial neural networks, which present a very similar performance for Train and Test datasets.

For this reason, ANN was the selected method to be used in our system, and the confusion matrix for this method is presented in Table 12. The class accuracy is also shown in Table 12, indicating that the most challenging fault for classification purposes is shadowing. This behavior can be justified by the great variability in the shadowing effects, since each event may present a great variability in the effects that were observed in the monitored variables. The overall accuracy is 90.05%.

### 7.4. Combination of Fault Detection and Classification

By combining the proposed fault detection method with the best classifier (ANN), as presented in the previous Section, the final confusion matrix is presented in Table 13. In order to obtain such result, first, instantaneous data are applied to the fault detection model and, for each detected fault, the classifier is used to identify the corresponding fault. With that, the confusion matrix includes the normal class.

The average class accuracy for Table 13 is 92.64%. When compared with the individual accuracy from detection (93.09%) and classification (95.44%) methods, one can observe that the result is inferior for the combined approach. However, it is similar to the result obtained for the detection stage, which demonstrates that most of the detected faults present high classification accuracy. The class with the most misclassifications is shadowing, i.e., 77% of individual accuracy, around 15% inferior when compared to the second class with more misclassifications (degradation). This is mainly due to the fact that it is the fault with more parameters that can vary, since it can occur in different ways, with different intensities, and in different amounts for each cell. Additionally, most of the observed errors in Table 13 are related to normal class, which indicates that this is the influence of the detection stage. The best class accuracy, on the other hand, is obtained for open-circuit, since it is the fault that causes the largest voltage and current drop and it cancels the generation contribution of an entire string.

### 7.5. Comparison with State-of-the-Art Methods

There are several related works that focus on detection and classification of faults in PV systems, as presented in Section 2. The lack of a standard protocol to generate and analyze faults, besides the absence of a public dataset, hampers a proper comparison between proposed method and related works. However, by selecting works that detect and classify similar faults, we can present a comparison with state-of-the-art models.

The recent works from [39,40] present a similar two-stage architecture when compared with this work. Additionally, they use auto regressive models to estimate the expected power output as a function of current environmental conditions. The difference lies in the fault detection methods, which makes the works complimentary. While [39,40] uses fuzzy inference models yielding 98.2% accuracy with 16 combinations of shadowing, short circuit and open circuit, they cannot operate without disturbing the normal operation of the system, disconnecting the whole system to evaluate VxI curves or run the tree search algorithm.

The proposal from [42,45], on the other hand, works when the PV modules are normally operating. The first achieved 100% accuracy when detecting short circuit and open circuit faults while the second yielded overall accuracy of 97.52% for the same faults. This way, we observe a similar performance for the mentioned faults when compared to our proposed methods, which yields 97.22% accuracy for short-circuit and 98.78% for open circuit faults.

Moreover, we argue that our work is complementary to the mentioned since our proposed system is installed in a plant that generates more power (5 kW vs 1.8 kW), is installed in a region with more panel temperature variation (40 ${}^{{}^{\circ}}$C vs 7 ${}^{{}^{\circ}}$C) and can detect Shadowing and Degradation faults besides different short/open circuit conditions. In addition, we can achieve 92.64% of overall accuracy (detection and classification) while presenting some advantages that can be summarized, as follows:

- it allows real-time fault detection and classification (detection and classification are performed every second), keeping the PV plant in operation (without disconnection);
- shadowing events are caused by real shadowing, which makes it difficult to characterize, unlike the controlled shadowing presented in [11,38] that normally increases the performance for that class; and,
- it presents a comparable performance to other works, notwithstanding those works use different databases and classification procedures.

### 7.6. Monitoring System

**Figure 14:**
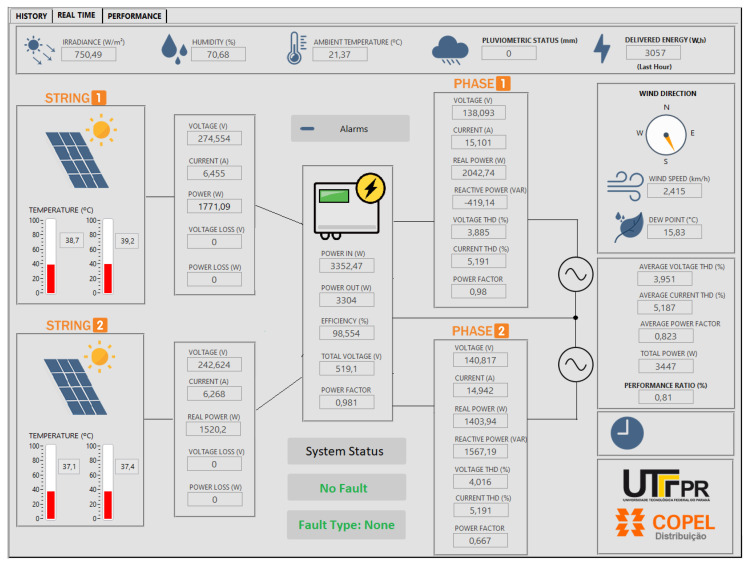
Human Machine Interface (HMI) Screen showing the real time measurements and the current status of the monitored PV plant.

Finally, this section describes the implemented system that collects all of the data to make them available in an execution environment to run the fault detection and fault classification algorithms. The calculated results are indicated as the current status of the PV plant. Figure 14 shows one of the HMI screens, namely the real-time tab, in which all instantaneous and integrated monitored signals, both electrical and environmental, can be viewed together with the system inferred status. The data disposition follows the generation on the PV strings in the left, the inverter and its parameter in the center, and the output of two phases at the right. The upper and right hand strips show the environmental conditions and performance. In this tab, pre-built and user configurable alarms can also be set. The HMI also implements a performance evaluation tab and a history tab, in which a configurable graph of the chosen signals can be plotted in a chosen time window. Because the system is a test bench for monitoring strategies, further details are reported in other work [8].

Regarding time results of the detection and classification methods in the embedded system, the ARX model takes 0.25 ms to run and provide the detection result, whilst the ANN classifier takes 13.60 ms to inform the final classification, resulting in 13.85 ms for the whole process. Such a result is less than the 1s time resolution used in the detection and classification modules, allowing the proposed online identification. Additionally, since the detection stage is less time consuming compared to the classification, it can be executed inside the main loop (1 s), triggering the classification model only in the case of possible faults, releasing the embedded system to perform the other monitoring related tasks.

## 8. Conclusions

Maintaining continuous energy production in PV systems is a recurring subject in power utilities. It has attracted attention from the academic community, particularly in the context of proposing mitigation techniques and automatic analysis of possible production deviations in PV plants.

In this work, we presented a MS that provides electrical and environmental variables measurements, allowing to record instantaneous and historical data and estimate parameters that are related to the plant performance. Integrated to this MS, we proposed an online detection and classification of faults, such as: short-circuit, open-circuit, partial shadowing, and degradation. The complete system is installed in a 5 kW PV plant and it was validated when considering 16 days with faults in different conditions.

Regarding the fault detection, we proposed a recursive linear model to detect faults in the system, using irradiance on the PV panel as input signals and power as output. The accuracy of the fault detection was 93.09%, which is considerably higher than other models that are normally used in the literature, such as Single and Double-Diode. In terms of classification, different machine-learning-based methods were compared, and the best accuracy was observed for an ANN model, with 95.44%. Additionally, simulated (and validated) data were used to train machine learning models, allowing to generate different fault conditions, increasing the generalization of the models.

By combining detection and classification, the overall accuracy was 92.64%. Such a result can be highlighted as relevant, since other state-of-the-art methods present comparable performance and do not present the integration of a fault detection and classification approach with an embedded PV plant monitoring system, allowing the online identification and classification of different PV faults, real-time and historical monitoring of electrical and environmental parameters of the plant. In addition, by making our real dataset publicly available, we intend to contribute to standardizing methods for comparing fault detection and classification methods.

In the sequence of this work, the authors intend to include the analysis of multiple simultaneous faults, besides adding more typical faults that can occur in the system, such as MPPT fault and other types of short-circuits. As a final validation of the proposed models, the system will also be integrated in different PV plants, some with higher rated power and some installed in different geographical regions that are subject to different environmental conditions. This way, the generalization capabilities of our system can be further reinforced.

## Figures and Tables

**Table 1:** CS6U-330P Electrical Data.

DATA	Value
Optimum Operating Voltage	37.2 V
Open Circuit Voltage	45.6 V
Optimum Operation Current	8.88 A
Short Circuit Current	9.45 A
Module Efficiency	16.97%

**Table 2:** Fault Schedule for Real Dataset.

Start Time	Runtime [min]	Condition
11:30	10	Degradation
11:45	10	Open-Circuit
12:05	10	Short-Circuit
12:25	10	Degradation
12:40	10	Open-Circuit
13:00	10	Short-Circuit

**Table 3:** Correlation index between environmental variables with the power of each string.

Environmental Variables	Power ($P_{\mathrm{dc},1}$)	Power ($P_{\mathrm{dc},2}$)
Irradiance (*G*)	0.96	0.96
PV Temperature (*T*)	0.86	0.86
Dew Point (*D*)	0.41	0.41
Ambient Temperature ($T_{a}$)	0.38	0.37
Relative Air Humidity (*H*)	−0.28	−0.27
Wind speed ($W_{s}$)	0.28	0.28
Wind direction ($W_{d}$)	0.02	0.02

**Table 4:** Results for the proposed fail detection process.

Model	NRMSE
ARX	0.82
Single-Diode Model (SDM)	0.77
Double-Diode Model (DDM)	0.76
Hammerstein-Wiener Model (HWM)	0.84

**Table 5:** Confusion matrix for fault detection, based on simple-diode model and an adaptive threshold.

Fault Detector\System	Normal	Fault
Normal	251,045	58,208
Fault	85,897	120,808

**Table 6:** Confusion matrix for fault detection, based on double-diode Model and an adaptive threshold.

Fault Detector\System	Normal	Fault
Normal	279,465	29,788
Fault	141,251	65,454

**Table 7:** Confusion matrix for fault detection, based on ARX model and an adaptive threshold.

Fault Detector\System	Normal	Fault
Normal	298,641	10,612
Fault	25,047	181,658

**Table 8:** Confusion matrix for fault detection, based on Hammerstein-Wiener model and an adaptive threshold.

Fault Detector\System	Normal	Fault
Normal	223,562	85,691
Fault	93,634	113,071

**Table 9:** Fault detection results for the Diode, ARX, and Hamerstein models in photovoltaic systems.

Property	ARX [%]	Simple-Diode [%]	Double-Diode [%]	Hammerstein- Wiener [%]
Accuracy	93.09	72.07	74.81	65.24
Precision	87.88	58.44	69.09	54.70
Sensitivity	94.48	67.48	68.34	56.89
Specificity	92.26	74.51	79.20	70.48

**Table 10:** Validation Results.

Case	DC Power MAPE
Normal	2.59%
Short Circuit	0.57%
Degradation	0.35%
Shadowing	2.33%

**Table 11:** Classification Results.

Classifier	Train Accuracy	Test Accuracy	Optimal Parameters
ANN	97.19%	95.44%	21 Neurons
SVM	93.55%	94.59%	C = 25/γ = 5
kNN	86.14%	89.96%	k = 13
Tree	84.94%	70.85%	max depth = 17/max leaf nodes = 100

**Table 12:** Classification Confusion Matrix.

Class	Short-Circuit	Degradation	Open Circuit	Shadowing	Class Accuracy [%]
Short-Circuit	5960	1	4	34	99.35
Degradation	600	9664	24	83	93.18
Open Circuit	0	0	6024	0	100.00
Shadowing	10,892	8,864	65	164,490	89.26
**Average**					95.45

**Table 13:** Confusion Matrix for Combined Detection and Classification Approaches.

Class	Normal	Short-Circuit	Degradation	Open-Circuit	Shadowing	Class Accuracy [%]
Normal	299,540	18	5613	0	4082	96.85
Short-Circuit	167	5832	0	0	0	97.22
Degradation	123	583	9644	14	7	92.99
Open-Circuit	73	0	0	5951	0	98.79
Shadowing	25,605	9202	6920	1	142,583	77.36
**Average**						92.64
